# Improved prediction of severe thunderstorms over the Indian Monsoon region using high-resolution soil moisture and temperature initialization

**DOI:** 10.1038/srep41377

**Published:** 2017-01-27

**Authors:** K. K. Osuri, R. Nadimpalli, U. C. Mohanty, F. Chen, M. Rajeevan, D. Niyogi

**Affiliations:** 1 Department of Earth and Atmospheric Sciences, National Institute of Technology, Rourkela, Odisha – 769 008, India; 2 Department of Agronomy and Department of Earth, Atmospheric, and Planetary Sciences, Purdue University, West Lafayette, IN 47907, USA; 3 School of Earth Ocean and Climate Sciences, Indian Institute of Technology, Bhubaneswar, Odisha – 751 007, India; 4 National Center of Atmospheric Research, Boulder, Colorado 80301, USA; 5 Ministry of Earth Sciences, New Delhi – 110003, India; 6 State Key Laboratory of Severe Weather, Chinese Academy of Meteorological Science, Beijing, China

## Abstract

The hypothesis that realistic land conditions such as soil moisture/soil temperature (SM/ST) can significantly improve the modeling of mesoscale deep convection is tested over the Indian monsoon region (IMR). A high resolution (3 km foot print) SM/ST dataset prepared from a land data assimilation system, as part of a national monsoon mission project, showed close agreement with observations. Experiments are conducted with (LDAS) and without (CNTL) initialization of SM/ST dataset. Results highlight the significance of realistic land surface conditions on numerical prediction of initiation, movement and timing of severe thunderstorms as compared to that currently being initialized by climatological fields in CNTL run. Realistic land conditions improved mass flux, convective updrafts and diabatic heating in the boundary layer that contributed to low level positive potential vorticity. The LDAS run reproduced reflectivity echoes and associated rainfall bands more efficiently. Improper representation of surface conditions in CNTL run limit the evolution boundary layer processes and thereby failed to simulate convection at right time and place. These findings thus provide strong support to the role land conditions play in impacting the deep convection over the IMR. These findings also have direct implications for improving heavy rain forecasting over the IMR, by developing realistic land conditions.

Severe thunderstorms (STS) are resultant of vigorous localized convective activity leading to thunder, lighting, heavy rain, hail, and squalls; which sometimes also result in tornadoes that cause massive destruction along the path. Over the Indian monsoon region (IMR), the eastern India and Bangladesh regions are frequently affected by STS during pre-monsoon months (i.e., April and May). Typically 28 STS occur in this region during these two months. These storms generally move from northwest to southeastwards. These are often poorly forecasted^1^, however, modest improvements were seen with assimilation of atmospheric observations [Global Telecommunication System (GTS) and Doppler Weather Radar (DWR) etc.] in simulating various convective systems over the IMR^2^,^3^. This may be mainly because of lack of high-resolution mesoscale models along with better initial conditions, particularly, land surface characteristics, and thus, the focus of this study is to improve the prediction of severe thunderstorms by initializing high resolution realistic land surface conditions.

India has seen notable land use land cover changes (LULCC) due to agricultural intensification, and rapid urbanization. Changes in land characteristics change surface energy balance terms^4^ and thereby rainfall characteristics^5^,^6^,^7^. The increased LULCC have been cited as reasons for increased local/mesoscale rainfall activity and associated heavy rains and flood potential^8^. These mesoscale rains significantly change the underlying land surface characteristics, creating wet and dry regions. The traditional perspective regarding the monsoon has been that large scale conditions overwhelmingly dominate the convective environment and hence the climatological land state has been the defacto mode of running forecast models by the operational and research community. Further, even though it is recognized that the climatological soil moisture and soil temperature (SM/ST) data may not be representative of these local changes, it has been considered a secondary feature behind improvements to cloud convection processes for instance^2^,^3^,^9^. In this study, the hypothesis that the simulation of localized severe convection associated with pre-monsoon STS over the IMR can be improved by direct incorporation of enhanced SM/ST fields as surface initial conditions to mesoscale model is tested. Specifically the role SM/ST plays in modulating mesoscale convection is evaluated in the present study. This study seeks to understand if the feedback from land surface characteristics which controls the surface energy and moisture budgets, and ultimately modifies the heat and moisture fluxes within the planetary boundary layer (PBL) can drive the deep convective activity as has been noted in midlatitudes^10^. Recent studies^11^,^12^,^13^,^14^,^15^ suggested that the land surface heterogeneity and feedback could be influential on the convective structure of rain bearing systems and contribute to improve the model performance.

## Results and Discussions

### Verification of Land Data Assimilation System (LDAS) over Indian region

One of the challenges in conducting a land surface assessment study over India is the paucity of *in-situ* verification data. Developing a high resolution analysis from a LDAS therefore becomes important. For the LDAS, hourly 2 m temperature (T2) forcing from the Modern-Era Retrospective analysis for Research and Applications (MERRA) analysis is utilized. This is verified with tower observations from a research site at Kharagpur (22.31°N, 87.31°E) during monsoon (June-September) and thunderstorm period (April-May). The MERRA-T2 analysis showed some overestimation. The correlation and root mean square error (RMSE) of T2 is 0.76 and 1.9 °C, respectively. The average maximum and minimum temperatures of MERRA analysis are 39.5 °C and 25.6 °C, while observations show 38.9 °C and 23.5 °C. The mean diurnal variation of the MERRA-T2 analysis is reasonably correlated (r = 0.95) with observation and the RMSE is ~0.88 °C. The diurnal pattern of MERRA-based T2 also showed similar variability during 06–15 UTC when compared to the tower observations. The other important forcing used to drive the LDAS is the precipitation from Tropical Rainfall Measuring Mission (TRMM) rainfall analysis (TMPA). Results indicate that TRMM rainfall replicates observed station rainfall patterns reasonably well with some discrepancies in capturing peak rainfall activity. The standard deviation, and RMSE of TRMM rainfall for Kharagpur station is 1.17, and 1.3 mm during monsoon season and they are ~1.37 and 1.4 mm hr^−1^ in thunderstorm period. These values are consistent to those reported over the monsoon region^16^. Results for monsoon period are shown in Supplementary Figure S1.

**Figure 1:**
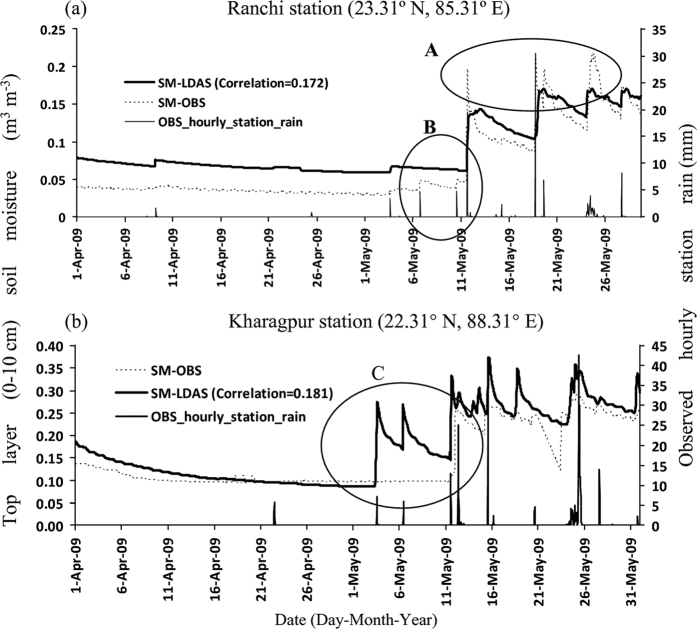
Validation of top layer (0–10 cm) soil moisture (SM, m^3^ m^−3^) obtained from LDAS with observed SM (same layer) at two meteorological tower stations (**a**) Ranchi (23.31°N, 85.31°E) and (**b**) Kharagpur (22.31°N, 87.31°E) for April and May months of 2009. Histograms (secondary y-axis) are observed hourly rainfall in mm. The figures are developed using Microsoft Excel in Office 2010 version.

From Fig. 1(a,b), the validation of hourly LDAS-based top layer (0–10 cm) SM shows good concurrence with the tower observations with some overestimation at Ranchi (23.31°N, 85.31°E) and Kharagpur (22.31°N, 87.31°E) stations during April-May of 2009. It can also be seen that the LDAS SM correctly responded to the observed rain (which is not used in LDAS forcing, see ‘method’ section) with few exceptions (ellipse A and B in Fig. 1a) during very heavy rainfall at Ranchi. The SM shows a strong correlation (0.97) with observed SM at Ranchi with standard deviation (SD) of SM error of 0.027 m^3^ m^−3^ (6%) and RMSE of 0.02 m^3^ m^−3^ (8%). The value in the parenthesis represents the percentage of error with respect to the SM saturation point (0.464 m^3^ m^−3^) of that station with silty clay loam category. At Kharagpur station, the LDAS-SM showed increased response corresponding to observed peak rainfall, while, the observed SM did not at times (elliptical C in Fig. 1b). The skill of LDAS-SM at Kharagpur is also good as noted by correlation of 0.89, error SD of 0.015 m^3^ m^−3^ (3.4%) and RMSE of 0.02 m^3^ m^−3^ (4.5%). Percentage of error is relative to soil saturation point (0.439 m^3^ m^−3^) of Kharagpur (with loam soil category). Similar statistics are noted at these two stations during monsoon period (June-September). The correlation (RMSE) of SM during summer monsoon at Kharagpur and Ranchi stations are 0.64 (0.03 m^3^ m^−3^) and 0.71 (0.05 m^3^ m^−3^) respectively. This analysis provides confidence about the performance of LDAS and its use as surface initial conditions in ARW model over the IMR.

### Case-1 (7 May 2010)

**Figure 2:**
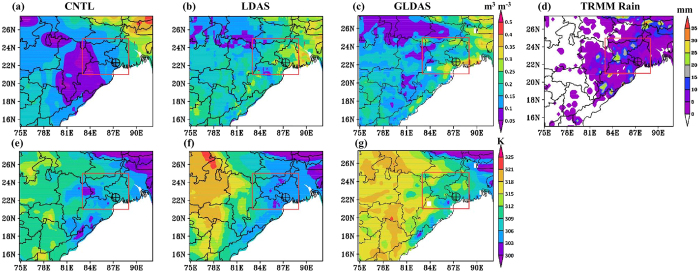
Spatial distribution of top layer (0–10 cm) SM (m^3^ m^−3^) from (**a**) CNTL and (**b**) LDAS and (**c**) GLDAS analyses valid for initial time, 12 UTC 6 May 2010. (**e**–**g**) are same (**a**–**c**) but for ST (K). (**d**) presents the previous 24 hr accumulated TRMM analyzed rainfall (mm) valid at initial time. Box represents the area of thunderstorm activity and the symbol represents the location of Kharagpur where it became severe. The figures are developed using open source software GrADS (Grid Analysis and Display System) version 2.1.0 (http://cola.gmu.edu/grads/downloads.php).

Figure 2 provides initial top layer SM and ST from both CNTL and LDAS analyses along with Global land data assimilation system (GLDAS) analysis for comparison. The prior 24 hr accumulated TRMM rainfall (mm) valid at the model initialization time showed ~25 mm rainfall in the region of thunderstorm activity marked by box in Fig. 2d. The wet and dry regions (high and low SM values) in LDAS-SM analysis are in coherence with the observed rainfall distribution. There is notable difference in CNTL-based SM (Fig. 2a) when compared to rainfall pattern (Fig. 2d). In case of GLDAS analysis, the thunderstorm region has SM of about 0.2–0.35 m^3^ m^−3^ with dry and wet patterns in the western and eastern parts respectively. The LDAS-based high resolution SM field showed a realistic local heterogeneity like in GLDAS analysis. Unlike GLDAS and LDAS, in CNTL analysis the regions of Orissa (O), Jharkhand (J) and some parts of Madhya Pradesh (MP) and West Bengal (WB) in the domain (refer Supplementary Figure S2 (g) for regions) are relatively drier (<0.1 m^3^ m^−3^) despite antecedent rainfall. There is false signal of heavy SM in northeast parts in CNTL run unlike in GLDAS and LDAS analysis. There is a west to east gradient in GLDAS-ST analysis from central India (75°E–82.5°E) to eastern India (82.5°E–92°E). This ST gradient is consistent with rainfall activity in the domain. This feature is clearly brought up by the LDAS analysis unlike CNTL analysis.

**Figure 3:**
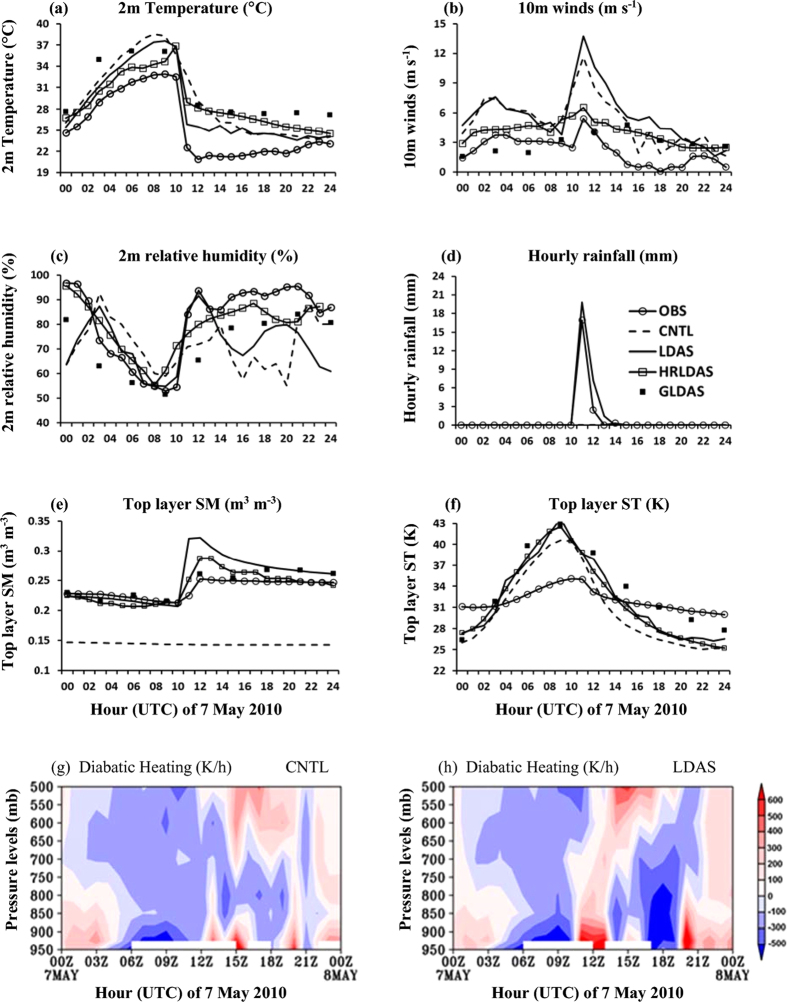
Hourly comparison of model parameters (**a**) 2 m temperature (**b**) 10 m winds (**c**) 2 m relative humidity (**d**) rainfall (**e**) top layer SM and (**f**) ST from CNTL and LDAS runs with Kharagpur tower observations for case-1 (7 May 2010). (**g**,**h**) Are time-height cross section of diabatic heating, (K/h) averaged over 1° × 1° around Kharagpur station from CNTL and LDAS respectively. The figures (**a**–**f**) are developed using Microsoft Excel in Office 2010 version and (**g**–**h**) using open source software GrADS (Grid Analysis and Display System) version 2.1.0 (http://cola.gmu.edu/grads/downloads.php).

Observations and field report indicate that the initial convection developed in the northwest of Dhanbad (Jharkhand) at 06 UTC 7 May 2010. While moving in southeastward direction, it intensified and became severe thunderstorm at Kharagpur (West Bengal) by 10 UTC. Time series of model simulated variables from CNTL and LDAS during thunderstorm day is compared with those of tower observations, high resolution land data assimilation system (HRLDAS) and GLDAS products and is presented in Fig. 3. Note that SM and ST from HRLDAS in Fig. 3(e,f) are integration-output and T2, relative humidity (Rh2), 10 m winds in Fig. 3(a,b,c) are inputs to HRLDAS from MERRA analysis. From Fig. 3, observations and analyses indicate that the surface parameters quickly (within one hour) change after 10 UTC with the thunderstorm. Between 10 and 11 UTC, there is 10 °C drop (from 32 to 22 °C) in T2, 3 m s^−1^ increase in 10 m wind speed, and 30% increase (from 54 to 84%) in Rh2. The CNTL run shows high temperatures, low moisture, and associated differences. LDAS run could capture these changes, showing 11 °C drop (from 36 to 25 °C) in T2, 26% rise in Rh2 (from 59 to 86%) at Kharagpur and is in close agreement with other analyses. CNTL run could not capture rainfall at Kharagpur station and hence SM shows little to no change. The LDAS initialized run reproduced rainfall of ~20 and 7 mm at 11 and 12 UTC respectively which is close to observation (17 and 2.4 mm). This rainfall contributed for the increase in top layer SM as seen in observations, LDAS and GLDAS and HRLDAS analysis. The observed diurnal variation of ST is less as compared to that of the model simulated, however, GLDAS and HRLDAS analysis showed similar diurnal variation of ST as in both runs. The land surface is relatively warmer in LDAS than CNTL run.

Supplementary Figure S3 shows the SM, ST and 2 m specific humidity (g kg^−1^) at 06 UTC 7 May 2010 (a 18 hr model forecast) valid for thunderstorm initiation. The land surface in CNTL run is not moist in the thunderstorm area (i.e. Jharkhand and West Bengal), while, the LDAS simulated moist region. The distribution of ST is similar in both the runs. As expected, the evapotranspiration values increase when the SM and ST are abundantly available and help increase the atmospheric moisture in the boundary layer^17^. LDAS simulated atmosphere is more unstable with high convective available potential energy (CAPE) and atmospheric precipitable water which support convection to initiate and maintain. From Fig. 3(g,h), the time-vertical cross section of diabatic heating (averaged 1° × 1° region around Kharagpur station) in CNTL run, showed cooling from ~5 UTC to 11 UTC. At and after 12 UTC the diabatic heating is confined to the lower levels (Fig. 3g). On the other hand, in the LDAS SM initialized run due to realistic surface boundary conditions, the evolution of diabatic heating is more realistic and consistent from ~10 UTC to 13 UTC and extended up to 500 hPa level (Fig. 3h). This heating through the middle atmosphere helps generate the low level positive potential vorticity which modifies the mass field and advection patterns in the lower levels and supports deep convection^18^. The area of positive vorticity is associated with strong updrafts (~180 cm s^−1^) in LDAS run, while, it is weak (~120 cm s^−1^). This temporal analysis shows that the convection in CNTL run seems to be delayed (after 12 UTC) and it is almost at same time in the LDAS run.

**Figure 4:**
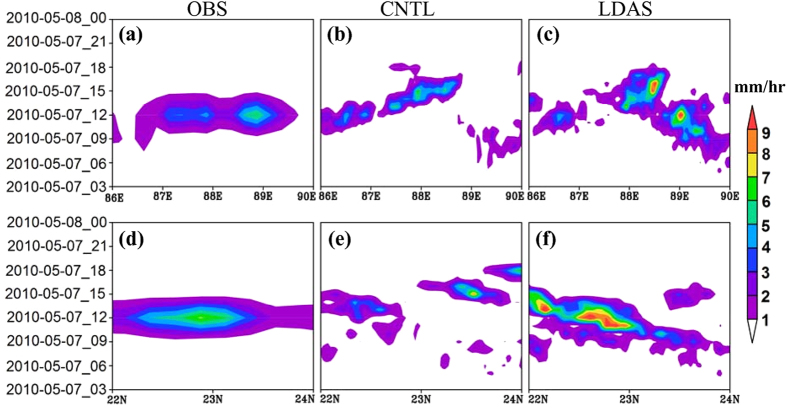
Time-longitude cross section of average rain rate (mm hr^−1^) between 21°–24°N (**a**) TRMM rainfall (**b**) CNTL and (**c**) LDAS. (**d**–**f**) Are same as (**a**–**c**) but for time-latitude cross section averaged between 87°–90°E for Case-1 (7 May 2010). The figures are developed using open source software GrADS (Grid Analysis and Display System) version 2.1.0 (http://cola.gmu.edu/grads/downloads.php).

Figure 4(a–c) shows temporal-longitudinal evolution of hourly rain rate (mm hr^−1^) averaged over 21°–24°N from TRMM, CNTL and LDAS respectively. Reviewing the TRMM fields, the rainfall (2–3 mm hr^−1^) started after 09 UTC around 86.5°–89.5°E and became maximum (6–7 mm hr^−1^) at 12 UTC around 88°–89°E. Though CNTL simulated rainfall is up to 6 mm hr^−1^, there is delay in the occurrence (Fig. 4b) as compared to the observations (Fig. 4a). The peak rainfall around 89°E is not simulated in the CNTL run. In case of LDAS run, both the spatial pattern and timing of the simulated rainfall is improved and is closer to observation (with peak rainfall of 9 mm hr^−1^ at 11–12 UTC around 89°E). The time-latitudinal observed rainfall (averaged between 87°–90°E) is confined between 9–15 UTC with peak rainfall around 12 UTC at 23°N (Fig. 4d). The LDAS run (Fig. 4f) reproduced more realistic rainfall pattern with some overestimation in rainfall amount, unlike CNTL (Fig. 4e). This analysis also suggests the misplacement of rainfall associated with convection in CNTL. These results highlight the importance of land surface conditions in rainfall prediction. Niyogi^13^ show how the accurate simulation of gradients in the surface fluxes can create the mesoscale boundaries that can have a significant impact on the rainfall intensity and distribution.

### Case-2 (12 May 2009)

**Figure 5:**
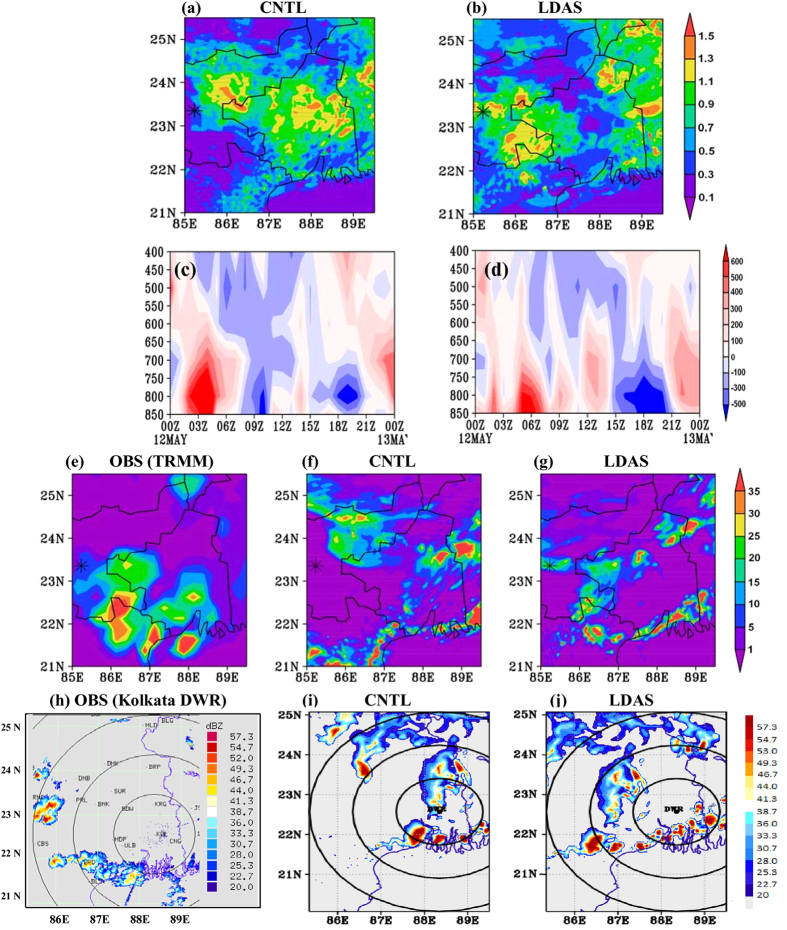
Model simulated surface moisture flux (kg m^−2^ s^−1^) from (**a**) CNTL and (**b**) LDAS averaged for 9–12 UTC 12 May 2009 (case-2). (**c**,**d**) Are same (**a**,**b**) but time-vertical cross section of diabatic heating (K/h) averaged over the region 86°–88.5°E and 21.5°–24°N. 3 hr (9–12 UTC) accumulated rainfall in mm from (**e**) TRMM (**f**) CNTL and (**g**) LDAS. (**h**–**j**) Are, respectively, the Kolkata DWR observed reflectivity, CNTL and LDAS simulated reflectivity (dBZ) at 11 UTC. The star in (**a**,**b**,**e**–**g**) represents the Ranchi station. The figures are developed using open source software GrADS (Grid Analysis and Display System) version 2.1.0 (http://cola.gmu.edu/grads/downloads.php). Figure (**h**) was shared by Radar division of India Meteorological Department as a part of data collection for STORM field experiment and there is no restriction in using this image.

The analysis of initial SM/ST of CNTL run for case–2 also showed similar patterns as in Fig. 3. Observations indicate, convection was initiated in the northwest part of Ranchi at 06 UTC and intensified into east-west oriented squall line over Orissa while moving in south-east direction and dissipated by 13 UTC. The time-averaged surface moisture flux (06–13 UTC 12 May 2009) is analyzed (Fig. 5a,b). Surface moisture flux is considerably high in the region of thunderstorm/convection genesis i.e, around Ranchi, in case of LDAS experiment with the availability of sufficient SM, and, is oriented from northwest to southeast direction (Fig. 5b). While, in CNTL run, it shifted to higher latitudes and dispersed along east-west direction (86°E to 89°E along 23°–24°N latitudes) (Fig. 5a). Therefore, the movement of thunderstorm activity is more realistic in case of LDAS run with the accurate representation of SM distribution. Krishnamurti^19^ demonstrated that the asymmetries in SM and its spatial variability create a spread of buoyancy elements and system is continually being steered to the new buoyancy regions. Analysis of instability parameters such as CAPE and precipitable water indicates suitable environment for the occurrence and intensification of severe convection in LDAS run. Observed CAPE and precipitable water at Ranchi is about 867 J kg^−1^ and 32 mm at 00 UTC and 4551 J kg^−1^ and 46 mm at 12 UTC respectively. The LDAS based CAPE values at 00 and 12 UTC are 1132, 4279 J Kg^−1^ and precipitable water is 28, 42 mm. These values are much underestimated (2878 J kg^−1^ and 31 mm at 12 UTC) in CNTL run. The intensification of the convection in LDAS run is also characterized by the increased mass flux^20^ in the boundary layer during ~6–14 UTC.

It is again notable that the LDAS could produce realistic diabatic heating in the boundary layer (Fig. 5d). While, the CNTL experiment exhibited poor mass flux evolution and diabatic cooling in the boundary layer during thunderstorm event i.e., 6–13 UTC 12 May 2009 (Fig. 5c). The differences in the distribution of diabatic heating are primarily attributable to differences in model-simulated moist convection and the associated clouds. The 6 hr accumulated rainfall (6–12 UTC 12 May 2009) from TRMM analysis indicates that majority of the rainfall is located over SE parts of Jharkhand, north Orissa and coastal West Bengal (Fig. 5e) which is also true for LDAS run. The CNTL run failed to capture the occurrence of rainfall as observed and showed false rainfall epochs over Orissa and west Bengal (Fig. 5f). However, the LDAS run could improve the model performance, meaning it eliminated the false rainfall signals (above 24°N) that appeared when model is initialized with climatological SM/ST fields. The model simulated station rainfall (>10 mm only) is compared with the ground-truth (IMD) rainfall (Supplementary Table ST1). The LDAS simulated rainfall is more realistic over most of the stations as compared to that of CNTL run. Though the CNTL run captured the peak rainfall (intensity-wise), it is misplaced location-wise due to unrealistic movement of the system. It can be noted that the TRMM rainfall underestimates actual rainfall in the study domain. The model-simulated reflectivity from both CNTL and LDAS runs at the time of peak thunderstorm activity (11 UTC 12 May 2009) is presented in Fig. 5(i,j) along with the observed reflectivity (which is at 1038 UTC, Fig. 5h). The observed reflectivity clearly shows east-west oriented convective cells and development of new/another convection cell over/around Ranchi station (23.35°N, 85.23°E). East-west pattern of convective cells is reproduced by the LDAS experiment as compared to CNTL run. However, both runs could not show development of new convective cell over Ranchi. Both CNTL and LDAS showed some false signals of severe convection especially around regions of complex topography. Clearly the better land surface initial fields enhance the model simulations over the study domain.

### Case-3 (27 May 2007)

The initial SM is improved in the LDAS fields as seen in case of Case-1. The more realistic distribution of moisture flux and convective updrafts, as seen in previous cases, impacted the distribution of atmospheric wind and moisture profiles in the domain. The simulated atmospheric soundings of zonal, meridional winds (m s^−1^), mixing ratio (g kg^−1^), and equivalent potential temperature (θ_e_, K), in the boundary layer (up to 700 hPa) at 12 UTC 27 May 2007 are compared at stations that observed thunderstorms: Kolkata (22.65°N 88.45°E), Bhubaneswar (20.25°N, 85.83°E), Ranchi (23.31°N, 85.31°E), and no-thunderstorm station Gauhati (26.10°N, 91.58°E). The results are summarized in Supplementary Figure S4. The run initialized with LDAS fields, the mass fields (mixing ratio and θ_e_) right up to 700 hPa at all the stations match the observations when compared to those of CNTL run. In case of wind fields, LDAS performance is modesty better. At the Gauhati station (S4 d), the CNTL and LDAS profiles are similar and close to the observed profiles up to 850 hPa inferring that both experiments could capture the non-thunderstorm event.

**Figure 6:**
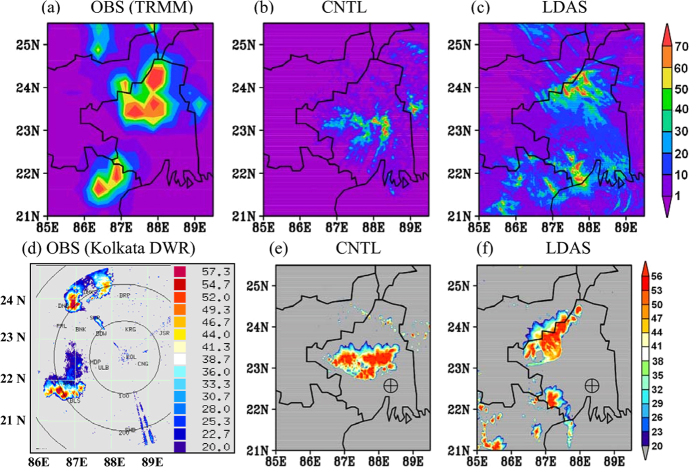
Model simulated 6 hr accumulated rainfall in mm (9–15 UTC 27 May 2007) from (**b**) CNTL and (**c**) LDAS along with (**a**) TRMM rainfall for case-3. (**d**–**f**) Are, respectively, the Kolkata DWR observed reflectivity, CNTL and LDAS simulated reflectivity (dBZ) at 11 UTC of same day. The crosshair symbol represents Kolkata DWR station. The figures are developed using open source software GrADS (Grid Analysis and Display System) version 2.1.0 (http://cola.gmu.edu/grads/downloads.php). Figure (**d**) was shared by Radar division of India Meteorological Department as a part of data collection for STORM field experiment and there is no restriction in using this image.

The 6 hr accumulated model-simulated rainfall during the thunderstorm activity (09–15 UTC 27 May 2007) is presented for case-3 in Fig. 6. According to observation (Fig. 6a), rainfall occurred in the north West Bengal and north Orissa states. The distribution and amount of rainfall is significantly improved in the LDAS (Fig. 6c) and replicate the observations. The CNTL run predicted rainfall in the central West Bengal and failed to predict rainfall over north Orissa. It is also noticed that the LDAS experiment could simulate even 1 hr accumulation of rainfall close to observed rainfall. The reflectivity analyses at 11 UTC (Fig. 6d–f) indicate that the LDAS run could simulate the strong convective echoes (of >56 dBZ) around ~87.5°E and ~22.2°N and 87–88°E and 23.5–24.5°N with less spatial error when compared with that of observed by the Kolkata DWR (Fig. 6d). The CNTL experiment predicted the convection in the central West Bengal (Fig. 6e). The LDAS could also succeed to simulate the northeast-southwest orientation of strong convection which showed a high degree of agreement with the observations (Fig. 6f). The LDAS simulated surface moisture flux and convective updrafts reinforce the atmospheric to be more suitable to initiate and intensify the convection in the right place.

### Statistical verification of model forecast

The verification analysis (see ‘method’ section) uses 68, 70, 74, 71, 85 and 83 observations in each case respectively. Mean error statistics indicate that the CNTL run underestimated the dew point temperature (Td) and relative humidity (Rh) by ~2 K and ~12% with a root mean square error (RMSE) of 8 K and 23% respectively. The performance is better in LDAS with a lower bias of 1 K and 8% and RMSE of 6 K and 18%, respectively. The surface Rh is incidentally under predicted in both the CNTL and LDAS experiments which can also be noted in Fig. 3.

**Figure 7:**
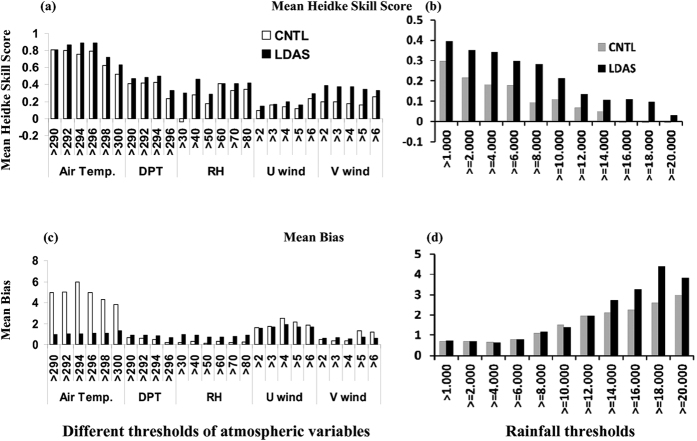
Mean Heidke skill scores at different thresholds of (**a**) atmospheric variables in boundary layer (950–700 hPa) valid for 24 hr forecast and (**b**) 6–12 UTC accumulated rainfall (cm) during thunderstorm day from CNTL and LDAS experiments. (**c**,**d**) Are same as (**a**,**b**) but for mean bias. [Air temperature, K; DPT = Dew point temperature, K; RH = Relative humidity, %; U wind = zonal wind, m s^−1^; V wind = Meridional wind, m s^−1^]. The figures are developed using open source evaluation tool (MET version 5) developed by NCAR (www.dtcenter.org/met/users/downloads/indes.php).

From Fig. 7(a,c), the categorical performance analysis demonstrated that the LDAS runs show high skill scores for all the thresholds of the variables compared to those of CNTL. Notable decreases are observed in the skill scores from smaller to higher thresholds in the CNTL run while the LDAS shows high skill scores even for higher thresholds. Compared to skill scores of mass fields, the wind skill scores are lower in both experiments, indicating the difficulty in wind prediction over the study domain. The LDAS show modest improvement in zonal wind skill scores although it is relatively better for meridional wind component. The improved meridional winds as a result of better simulation of divergence fields enhance moisture loading in the boundary layer and appeared to have aided the simulation of unstable environment in LDAS experiment. LDAS shows higher skill scores (from 0.4 to 0.8) and less bias (less than 3) for mass fields (temperature, Td and Rh). In the CNTL case, except for air temperature, the skill score is less than 0.5 for Td and Rh (and even negative for 30% Rh threshold). Rainfall skill scores shown in Fig. 7(b,d) indicate that LDAS run showed higher skill (>0.2) for up to 100 mm rainfall and thereafter decreased. The higher skill score for up to 60 mm rainfall in CNTL run decreased considerably for higher rainfall thresholds (became negative for rainfall amounts greater than 160 mm). The bias is almost similar for both the runs up to 12 cm rainfall threshold. Thereafter, the bias in LDAS is slightly increased and may be due to the overestimation of rainfall as compared with TRMM rainfall.

## Conclusions

Based on the above results and synthesis, the following conclusions are drawn that have major implications for improving mesoscale severe convection and thunderstorm predictions over the Indian monsoon region (IMR).

The high-resolution land data assimilation system could provide more realistic land surface fields over the IMR. The HRLDAS-based SM showed overall strong correlation (~0.9) with observed SM. The verification though limited, provides sufficient confidence for using HRLDAS fields as surface initial conditions for mesoscale models (ARW) over the IMR.

Improved initial values of SM and ST profiles helped to improve the forecast fields of surface moisture fluxes and near-surface atmospheric conditions such as temperature, moisture, and wind fields. This in turn improved the mass flux and diabatic heating in the boundary layer through stronger convective updrafts. As a result, the LDAS could reproduce the observed features of severe convection in terms of strong reflectivity echoes. The location, timing, and movement of thunderstorms are also improved when the LDAS-SM and ST fields are initialized within the ARW model. Further, it could also simulate more realistic rainfall distribution and intensity in the right place and time. In contrast, the CNTL run could not simulate the severe convection at right place and time because of improper representation of SM/ST fields in the model initial state that produced inadequate dynamic and thermodynamics features in the boundary layer. Statistical analyses also indicated better simulation of atmospheric parameters, particularly, the qualitative and quantitative rainfall with high skill scores and less error.

The results from different cases provide ample evidence that the proper representation of land surface characteristics (like dry and wet soil boundaries through soil moisture and temperature fields) are important drivers for mesoscale moist convection over the IMR. These spatial heterogeneities can indeed interact with synoptic-scale processes which are crucial in developing local-scale deep convection. Enhanced representation of pre-storm land state conditions can thus help to improve model performance in the simulation of deep convection and rainfall associated with severe thunderstorms over the pre-monsoon environment.

Note that while the performance of LDAS is better compared to the CNTL experiment, there are some limitations in LDAS such as spatial error, missing or false convective cells and associated rainfall bands. Ongoing study under a project through the India National Monsoon Mission addresses this issue to improve the model performance further by improving the input forcing fields to the LDAS system for more realistic SM/ST and other land surface fields.

## Methods

Long-term high resolution gridded SM/ST observations do not exist at regional scales, particularly over the Indian monsoon region (IMR) and assimilation of point SM/ST observations in a high resolution mesoscale model is complicated because of spatiotemporal variations in land surface conditions such as topography, soil texture, and vegetation characteristics. One optional approach is offline-preparation of SM/ST gridded profiles with depth on the exact forecast-model grid points of the study domain. Such experiment over the IMR is of first kind, particularly, for assessing the impact on simulation of STS.

For this purpose, a High Resolution Land Data Assimilation System (HRLDAS)^21^, based on Noah land surface model (LSM) is utilized to prepare the SM/ST fields. To prepare SM/ST fields, the HRLDAS typically use observed/analyzed initial data (vegetation fraction and associated land cover characteristics, skin temperature, soil characteristics) and atmospheric surface forcing data such as 2 m temperature (T2) and specific humidity (Q2), 10 m winds, surface pressure, model elevation, total rainfall rate, downward short wave radiation at surface and, downward long wave radiation. These fields were derived from the MERRA reanalysis^22^. The MERRA fields are proven to provide better representation of the atmospheric conditions^22^. These fields were augmented with rainfall forcing derived from 3-hourly rain rate of Tropical Rainfall Measuring Mission (TRMM 3B42) version**-**7 analyses^23^ to complement the MERRA-based precipitation analysis over the IMR^22^. The MERRA-based precipitation analysis showed poor performance over the IMR^22^,^24^. The gridded TRMM precipitation analysis (mm hr^−1^) is available at 0.25° × 0.25° resolution. The 3-hour rain rate from TRMM is interpolated linearly to hourly rain rate. The quality of MERRA-T2 and TRMM-rainfall is verified at Kharagpur station with available meteorological tower data (Supplementary Figure S1) and discussed in the results section. The initial land surface fields are derived from global data assimilation system (GDAS) analyses of National Centers for Environmental Prediction (NCEP). The land surface characteristics are based on the 24-category land use and 16-category soil texture, green vegetation fraction available from United States Geological Survey (USGS). The HRLDAS output includes soil moisture, (SM, as volumetric water content in m^3^ m^−3^), soil temperature, (ST, K) and surface energy balance components such as latent, sensible and ground heat fluxes (W m^−2^). For this study, the HRLDAS was initialized on 1 January 2006 and run up to 30 June 2010. The first year output was used for model spin up requirements^25^.

For this study, six severe thunderstorm cases were identified. These events occurred on 16 May 2007, 22 May 2007, 27 May 2007, 12 May 2009, 15 May 2009, and 07 May 2010 over the eastern and northeastern parts of India, and Bangladesh during the field phase of the Severe Thunderstorm Observations and Regional Modeling (STORM) program. Representative results have been presented for three cases i.e. 7 May 2010 (Case-1), 12 May 2009 (Case-2), 27 May 2007 (Case-3), that is one case for each year of the STORM campaign. All three cases are initialized at 12 UTC of the previous day and the forecast saved in one hour intervals for 36 hours. Details regarding the synoptic conditions associated with these STS cases are found in the STORM campaign report^26^,^27^. A brief description is provided in Table 1. The Infrared cloud imageries at the time of peak thunderstorm activity from Indian satellite, Kalpana are shown in Supplementary Figure S2(a–c) for each representative case respectively and in (d–f) for remaining three cases. Supplementary Figure S2(g) shows the locations of the different places referred in the subsequent discussions.

The Advanced Research Weather Research and Forecasting (ARW)^28^ model is used to simulate STS over the IMR. Both HRLDAS and ARW are configured on the same grid structure and domain to aid seamless incorporation of the surface fields into the mesoscale model initial conditions. A single domain covering 15°N–27.5°N and 75°E–92°E with 3 km horizontal grid spacing with the domain centered at 21.25°N and 83.5°E is used. The model is setup with 51 terrain-following hydrostatic vertical pressure (σ) coordinates. The σ coordinates are placed close together in the lower atmosphere (12 levels below 850 hPa and 22 levels below 500 hPa) and are relatively coarser in upper atmosphere. The grid structure follows the Arakawa C-grid. The horizontal and vertical resolution was based on prior results involving thunderstorm simulations and the model performance^2^. The Mellor-Yamada-Janjic (Eta) scheme is used for planetary boundary layer physics with Monin-Obukhov (Janjic Eta) scheme as surface physics. Noah scheme with four layers is used for land surface model. RRTM and Dudhia schemes are used for longwave and shortwave radiation physics. WSM 6 microphysical parameterization is used with explicit convection.

The numerical experiments were conducted, first without and then with HRLDAS-based SM/ST data initialization. In the former experiment, known as CNTL, the FiNaL (FNL) analyses of NCEP are used as initial and boundary conditions. The land surface data such as soil texture, green fraction, vegetation, soil moisture, and temperature, and land use are obtained from USGS climatological values. This is the default approach typically adopted over the IMR for rainfall prediction studies^2^,^29^,^30^. In the second experiment (known as LDAS), the land surface conditions are initialized from the HRLDAS fields created for the experimental runs. Except for the incorporation of initial SM/ST fields, there are no other differences in both the experiments in terms of model configuration and physics. An additional run is conducted (for case-1 and case-2) with a nested domain of 3 km horizontal grid spacing within a 9 km parent domain (2°N–38°N and 60°E–102°E) to compare the performance of single domain versus nested domain runs (with CNTL configuration). The experiment enables to assess the impact of (i) downscaling the global fields to cloud-convection permitting scale (3 km) directly and (ii) downscaling first to mesoscale (9 km) and then to cloud-convection permitting scale (3 km). Supplementary Figure S5 shows that the rainfall patterns are comparable in both the runs. However, the nested domain runs exhibited higher rainfall amount, and spatial distribution is not improved. Earlier study^31^ also demonstrated that the simulations of the extreme weather events by the global to convective scale downscaling were superior to those involving a nesting by the global to regional to convective scale downscaling. It is to be noted that two nested-domain runs consumed more computational time with 32 processors in Linux server of Intel compilers (~3 times). A recent study^32^ demonstrated that doubling the horizontal grid space typically increases the integration cost by a factor of 8. Therefore because of improved model performance and due to the computational efficiency, single domain configuration is adopted for this study.

The model forecast of temperature, dewpoint temperature, relative humidity, and wind within the lower atmosphere at 7 vertical levels (950, 925, 900, 850, 800, 750, and 700 hPa), and rainfall (cm) is verified against observations provided by IMD using the National Centre for Atmospheric Research (NCAR) Model Evaluation Tool (MET^33^). The error metrics such as bias and RMSE, and, categorical skill score metrics such as the Heidke skill score (HSS) and bias at different thresholds of the above mentioned variables are computed at 12 UTC of thunderstorm day. 6 hr (6–12 UTC) accumulated rainfall is verified with TRMM rainfall for skills scores. For a perfect forecast, the HSS and bias will be 1. The negative HSS values suggest that the number of hits by chance are higher. A bias greater than 1 represents overestimation and less than 1 represents under prediction.

## Additional Information

**How to cite this article**: Osuri, K. K. *et al*. Improved prediction of severe thunderstorms over the Indian Monsoon region using high-resolution soil moisture and temperature initialization. *Sci. Rep.* **7**, 41377; doi: 10.1038/srep41377 (2017).

**Publisher's note:** Springer Nature remains neutral with regard to jurisdictional claims in published maps and institutional affiliations.

## Supplementary Material

Supplementary Figures

## Figures and Tables

**Table 1:** Overview on synoptic conditions associated with the thunderstorm cases.

Cases	Date of event	Model initial time	Synoptic situations during thunderstorm activity
Case-1	7 May 2010	12 UTC 6 May	An echo was noticed to the northwest of Dhanbad at 0540 UTC, which intensified into a NE-SW oriented squall line of 150 km length by 06 UTC. Moving southeastwards it further intensified about 600 km in length by 1052 UTC and dissipated over the sea by 1600 UTC. About 6 cm of 24 hr accumulated rainfall was observed in the thunderstorm region.
Case-2	12 May 2009	12 UTC 11 May	A strong echo noted to the NW of Dumka at 0010 UTC which moved eastwards and dissipated by 0600 UTC. Another echo developed 100 km NW of Kolkata at 0430 UTC which moved south eastwards and intensified into squall line oriented east west, it dissipated over sea by 1300 UTC. Fresh echo developed over Ranchi at 0923 UTC which moved SE and dissipated by 1400 UTC. WB and Odisha received about 80 and 50 mm rainfall during 24 hr respectively.
Case-3	27 May 2007	12 UTC 26 May	A strong echo developed to the west of Dumka at 09 UTC that moved in south-easterly direction. The echo further intensified at 11 UTC. At 13 UTC it was located as line squall (length 200 km with east-west orientation) at about 120 km to the NW of Kolkata. It moved slowly southeastwards and was over Kolkata by 14 UTC and dissipated afterwards. A total amount of observed rainfall during this day is about 50 mm in WB and surrounding regions.
Case-4	15 May 2009	12 UTC 14 May	Strong echoes developed near Dumka and Ranchi which intensified into a squall line by 1100 UTC, moving southeastwards dissipated over sea by 1700 UTC. According IMD observations, West Bengal (WB) and Odisha states received about 40 and 30 mm rainfall during 24 hr respectively.
Case-5	22 May 2007	12 UTC 21 May	A strong echo developed to the south of Bankura at 10 UTC and moved southeastwards. It dissipated within 50 km west of Kolkata at 17 UTC. Odisha received about 20 mm rainfall and northeast states received 180 mm on 22 May.
Case-6	14 May 2007	12 UTC 13 May	A strong echo which moved southeastward was developed to the west of Kolkata at 09 UTC and dissipated at 11 UTC. Another echo developed to the NW of Baripada at 08 UTC and dissipated at 11 UTC while moving southwards. Odisha and WB states received about 30–50 mm rainfall.
