# Supplementary material for: Improved prediction of severe thunderstorms over the Indian Monsoon region using high-resolution soil moisture and temperature initialization
Source: Sci Rep. 2017 Jan 27;7:41377. doi: 10.1038/srep41377 (PMC5269717; doi:10.1038/srep41377)
Supplement: Supplementary Figures [file srep41377-s1.pdf]

Supplementary Information for

Improved prediction of severe thunderstorms over the Indian Monsoon region using high resolution soil moisture and temperature initialization

Krishna K. Osuri[1, 5], R. Nadimpalli[2], U. C. Mohanty[2], F.Chen[3+], M. Rajeevan[4], and D. Niyogi[5*]

[1] Department of Earth and Atmospheric Sciences, National Institute of Technology, Rourkela, Odisha – 769 008, India

[1] School of Earth Ocean and Climate Sciences, Indian Institute of Technology, Bhubaneswar, Odisha – 751 007, India

[3] National Center of Atmospheric Research, Boulder, Colorado, USA

[4] Ministry of Earth Sciences, New Delhi – 110003, India

[5] Department of Agronomy and Department of Earth Atmosphere and Planetary Sciences, Purdue University, West Lafayette, IN 47907, USA

[+]State Key Laboratory of Severe Weather, Chinese Academy of Meteorological Science, Beijing, China

Key words: Soil moisture, Soil temperature, Land data assimilation system, high resolution, severe thunderstorms, mesoscale convection, Indian monsoon region

Nature Scientific Reports

[*]Corresponding Author:

Prof. Dev Niyogi,
Purdue University, West Lafayette, Indiana
niyogi@gmail.com

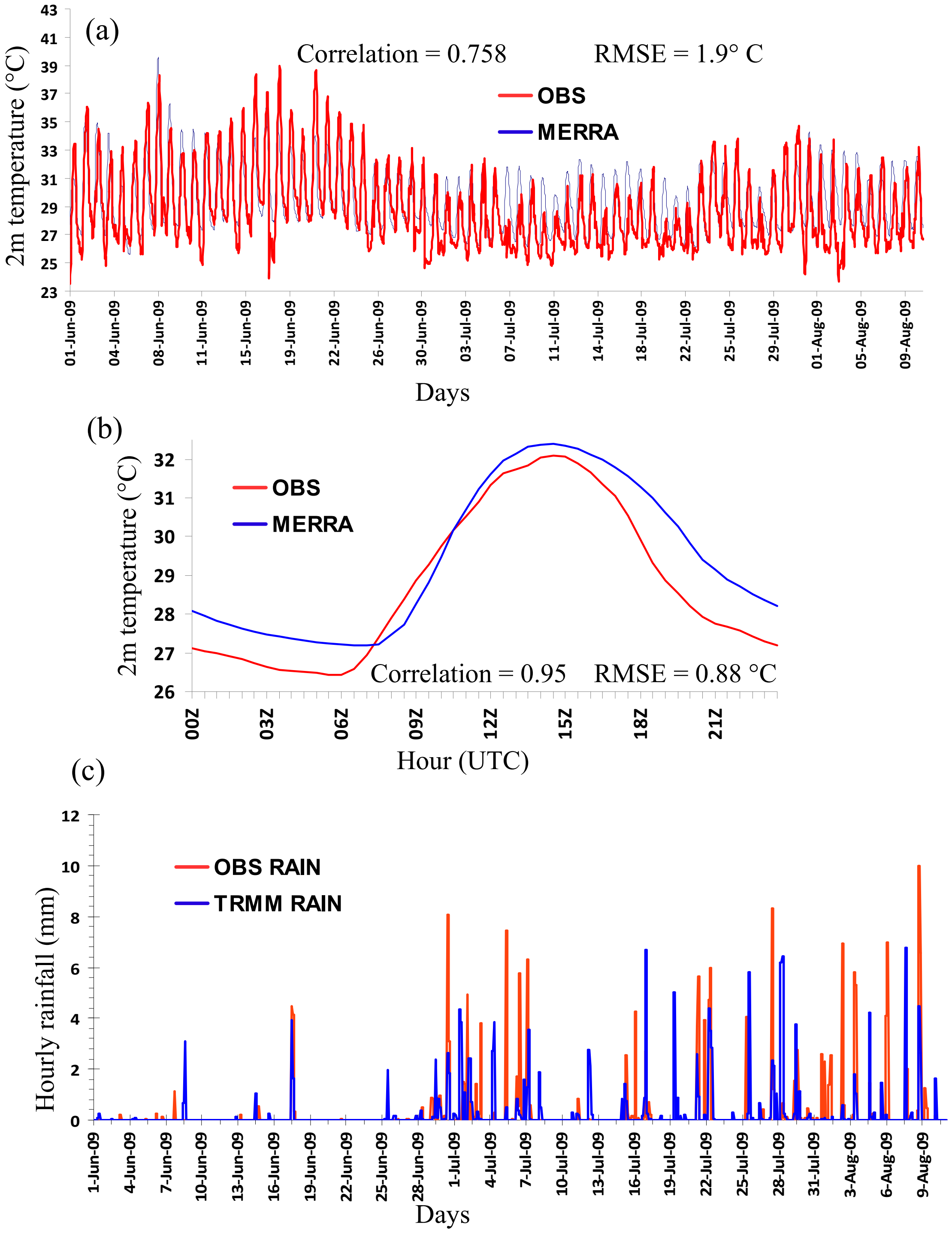

Supplementary Figure S1: Verification of HRLDAS forcing fields at Kharagpur station (22.31° N, 87.31° E) of (a) T2 from MERRA reanalysis from 1 June – 10 August 2009 (71 days) and (b) 71 day's mean diurnal variation of T2 and (c) TRMM rainfall analysis (mm) for the year 2009. The figures are developed using Microsoft Excel in Office 2010 version.

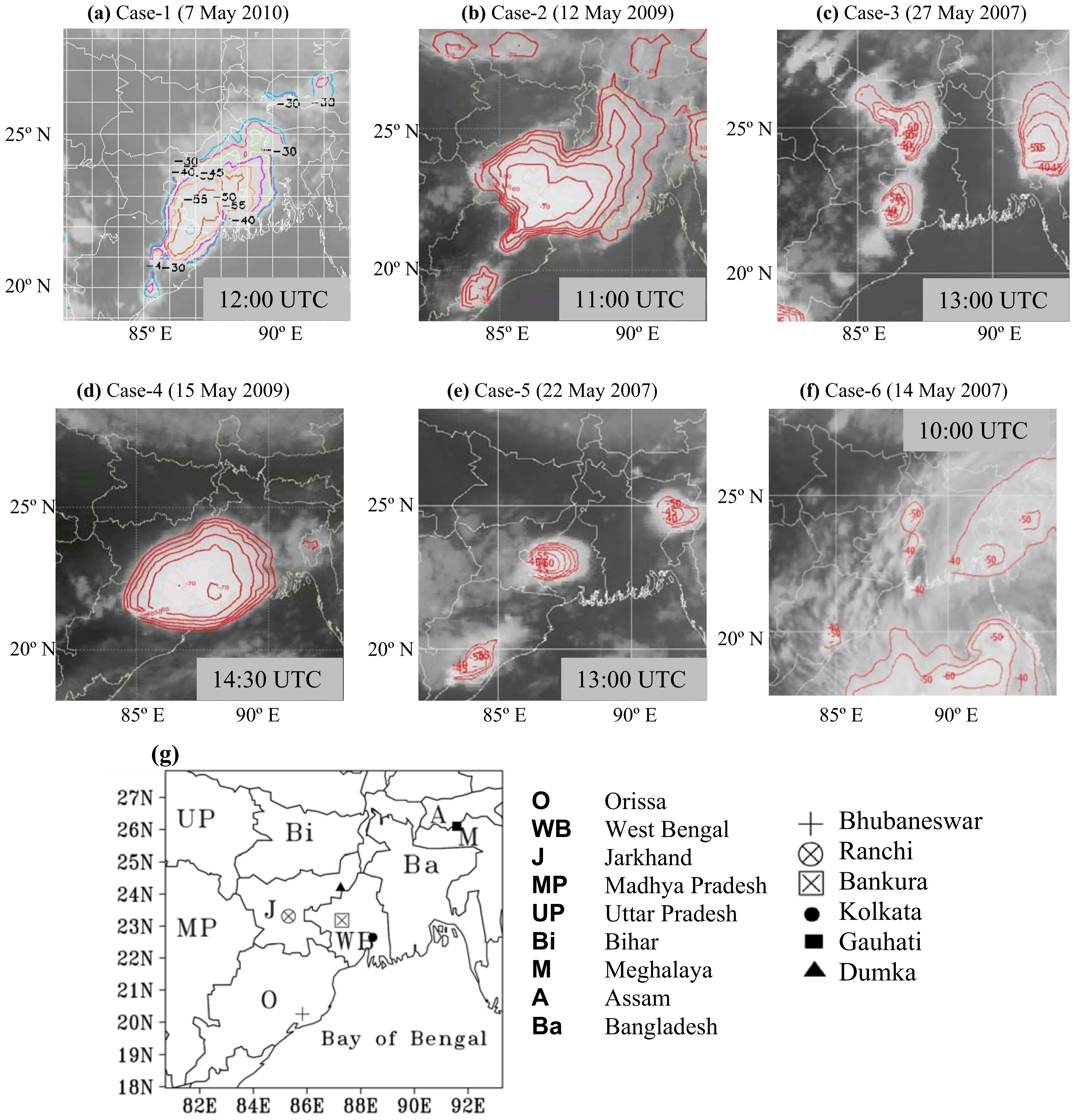

Supplementary Figure S2: Kalpana satellite visible imagery at peak thunderstorm activity (time in UTC is shown on each panel) for the six events (a) 7 May 2010 (Case-1), (b) 12 May 2009 (Case-2), (c) 27 May 2007 (Case-3), (d) 15 May 2009, (e) 22 May 2007, (f) 14 May 2007. (g) Location of important stations and names of states in India and Bangladesh referenced in the study. Contours represent cloud top temperature in °C in (a-f). These images were shared by satellite meteorological division of India Meteorological Department as a part of data collection for STORM field experiment. The data collected during STORM program was distributed freely to many organizations and individuals and there is no restriction in using these images. The figure (g) is developed using open source software GrADS (Grid Analysis and Display System) version 2.1.0 (http://cola.gmu.edu/grads/downloads.php).

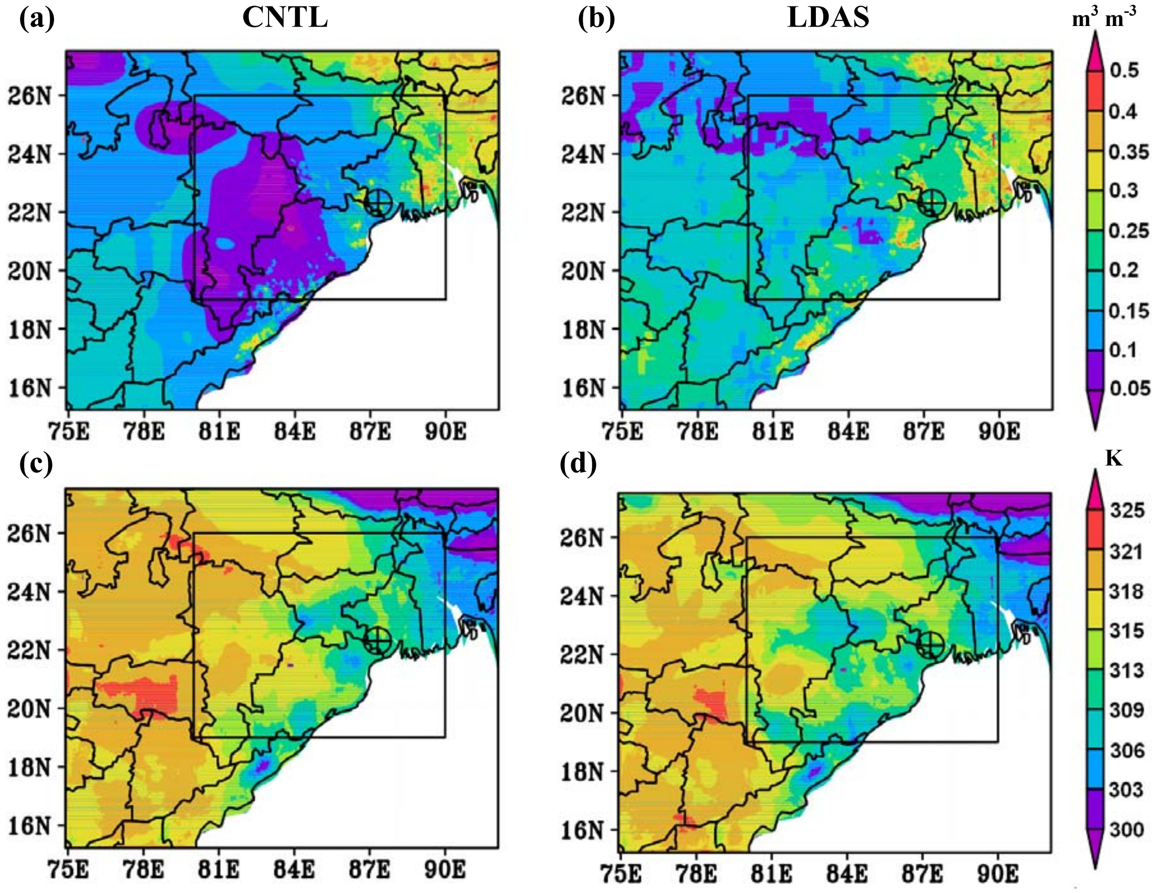

Supplementary Figure S3: Spatial distribution of top layer SM (m^3 m^{-3}) at the time of thunderstorm initiation, 06 UTC 7 May 2010 (at 19 hr model forecast) from (a) CNTL (b) LDAS runs for case-1. (c – d) are same as (a – b) but for ST (K) and (e – f) are same as (a – b) but for 2m specific humidity (g kg^{-1}) in the box shown in (a – d). The symbol represents Kharagpur station. The figures are developed using open source software GrADS (Grid Analysis and Display System) version 2.1.0 (http://cola.gmu.edu/grads/downloads.php).

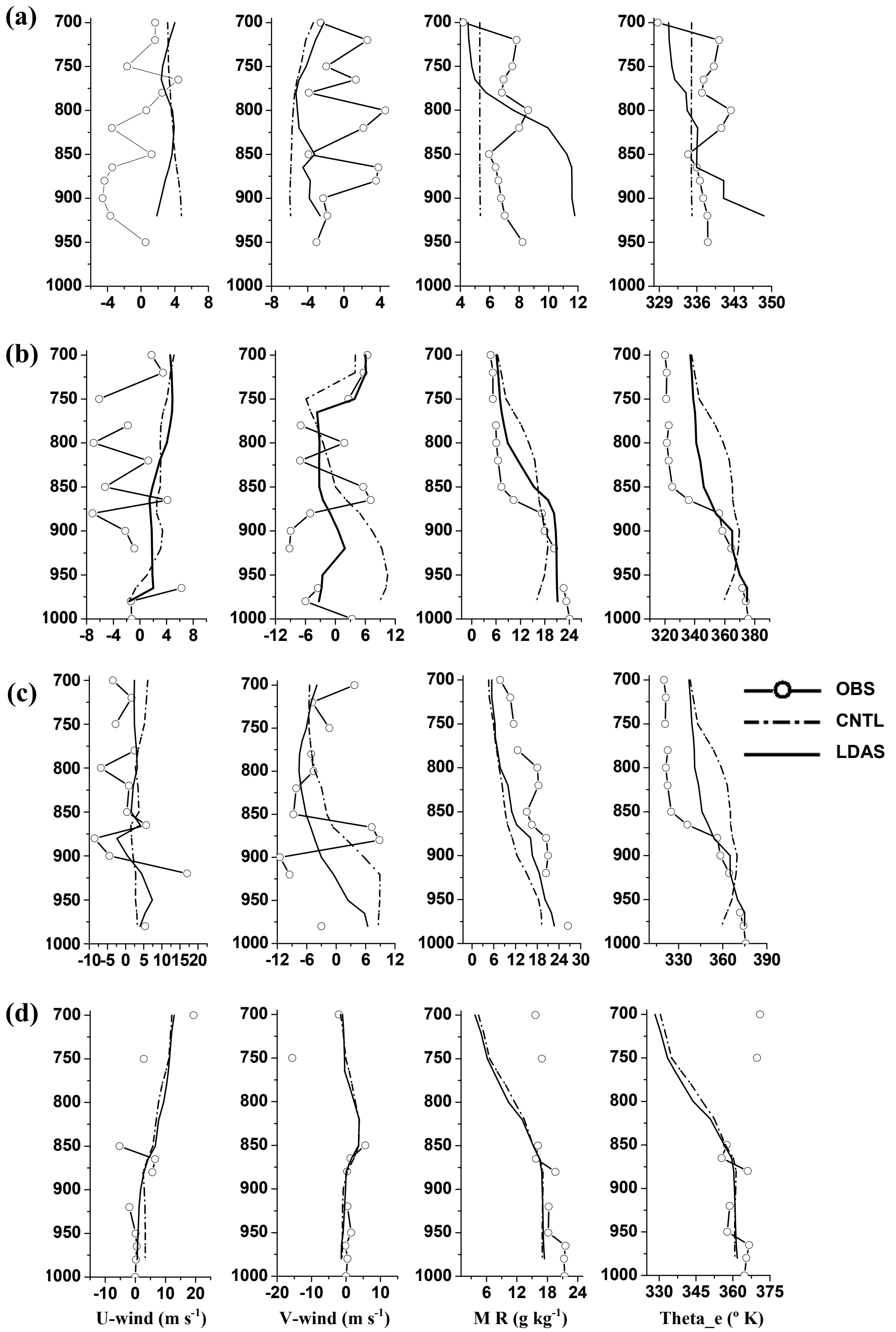

Supplementary Figure S4: 24-hr forecast of upper air sounding of zonal, meridional winds (m s^{-1}), mixing ratio, (g kg^{-1}) and equivalent potential temperature (K) corresponding to case-3 at (a) Ranchi, 23.31° N, 85.31° E (b) Kolkata, 22.65° N 88.45° E (c) Bhubaneswar, 20.25° N, 85.83° E and (d) Gauhati, 26.10° N, 91.58° E. The figure is developed using OriginLab software (http://www.originlab.com).

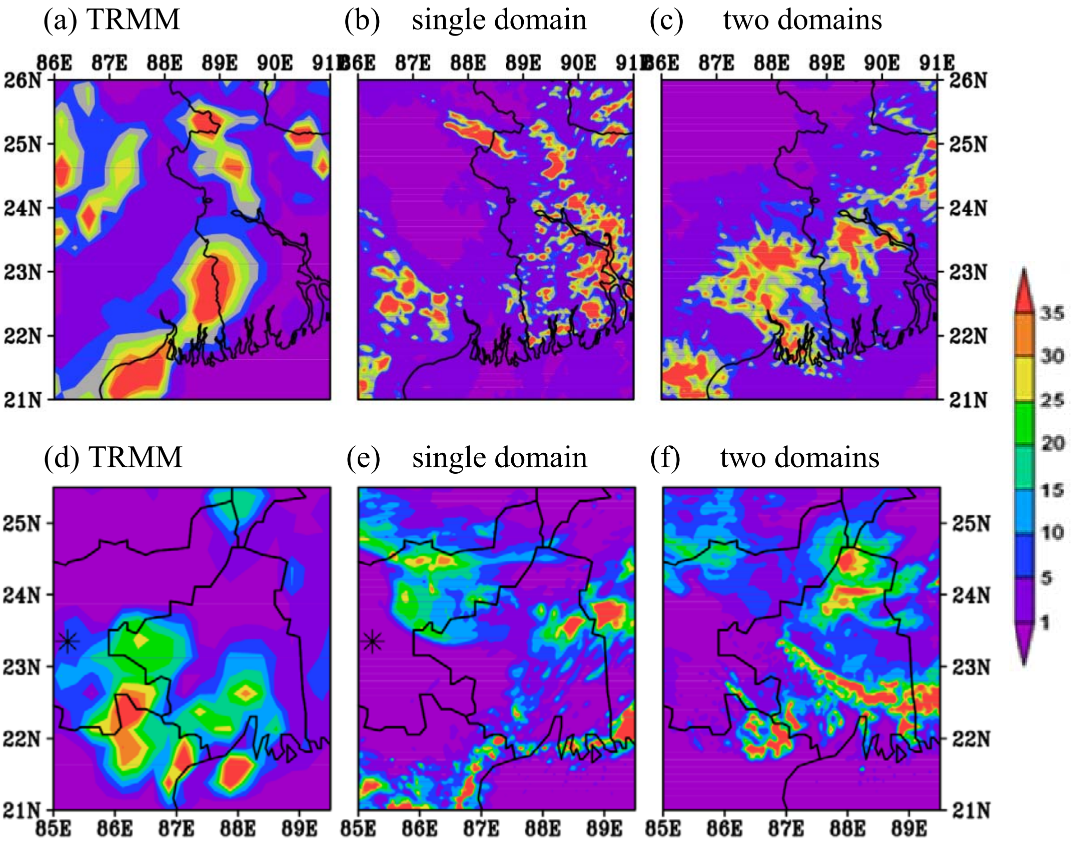

Supplementary Figure S5: 6-hr (06-12 UTC) accumulated rainfall for (a) TRMM (b) single domain and (c) two domain configurations during thunderstorm day of case-1 (top row). (d – f) are same as (a – c) but for case-2 (bottom row). The figures are developed using open source software GrADS (Grid Analysis and Display System) version 2.1.0 (http://cola.gmu.edu/grads/downloads.php).

Supplementary Table ST1: 24-hour accumulated station rainfall (mm) validation for case-2 (12 May 2009). Model-based data is extracted using open source software GrADS (Grid Analysis and Display System) version 2.1.0 (http://cola.gmu.edu/grads/downloads.php). Station observations are obtained from India Meteorological Department.

Station	Latitude	Longitude	IMD OBS	CNTL	LDAS
Sukiapokhri	22.99	87.85	38.4	13	25.5
Lava	27.09	88.67	18.3	14.5	20.4
Diana	26.85	89.00	11	5.7	10.6
Balurghat	25.22	88.77	17	10.2	15.6
Malda	25.01	88.14	31.3	3.6	22.6
Berhampur	24.10	88.27	27	10	25.2
Srinikethan	23.67	87.68	19.1	45.5	26
Uluberia	22.47	88.10	53.4	3.2	40
Alipore	22.53	88.33	12	2.2	7.3
Canningtown	22.31	88.66	15.6	1	10.4
Diamond Harbour	22.19	88.19	78.9	15	47.2
Digha	21.63	87.52	31.5	0.7	15
Haldia	22.06	88.11	43	14	31.7
Kalaikunda(IAF)	22.34	87.23	18	6.4	11
IIT, Kharagpur	22.31	87.31	38.7	13.2	27.4
